# Genes in pediatric pulmonary arterial hypertension and the most promising *BMPR2* gene therapy

**DOI:** 10.3389/fgene.2022.961848

**Published:** 2022-11-24

**Authors:** Lingling Dai, Lizhong Du

**Affiliations:** Department of Neonatology, The Children’s Hospital, National Clinical Research Center for Child Health, Zhejiang University School of Medicine, Hangzhou, China

**Keywords:** genetics, gene therapy, PAH, BMPR2, childhood-onset

## Abstract

Pulmonary arterial hypertension (PAH) is a rare but progressive and lethal vascular disease of diverse etiologies, mainly caused by proliferation of endothelial cells, smooth muscle cells in the pulmonary artery, and fibroblasts, which ultimately leads to right-heart hypertrophy and cardiac failure. Recent genetic studies of childhood-onset PAH report that there is a greater genetic burden in children than in adults. Since the first-identified pathogenic gene of PAH, *BMPR2*, which encodes bone morphogenetic protein receptor 2, a receptor in the transforming growth factor-β superfamily, was discovered, novel causal genes have been identified and substantially sharpened our insights into the molecular genetics of childhood-onset PAH. Currently, some newly identified deleterious genetic variants in additional genes implicated in childhood-onset PAH, such as potassium channels (*KCNK3*) and transcription factors (*TBX4* and *SOX17*), have been reported and have greatly updated our understanding of the disease mechanism. In this review, we summarized and discussed the advances of genetic variants underlying childhood-onset PAH susceptibility and potential mechanism, and the most promising *BMPR2* gene therapy and gene delivery approaches to treat childhood-onset PAH in the future.

## Introduction

Pulmonary arterial hypertension (PAH) is a rare but progressive and lethal vascular disease with the clinical definition of mean pulmonary arterial pressure >20 mmHg, normal left atrial pressure, and pulmonary vascular resistance ≥3 Wood units (Simonneau et al., 2019; Ruopp and Cockrill 2022). Histopathology of PAH demonstrates that the occlusion of occluded pulmonary arterioles results from the proliferation of endothelial cells, smooth muscle cells in the pulmonary artery, and fibroblasts, which ultimately results in right-heart hypertrophy and cardiac failure (Tuder et al., 2013). The estimated prevalence of childhood-onset PAH is about 4–16 cases per million, with a severe morbidity and high mortality (Li et al., 2017; Rosenzweig et al., 2019). The childhood-onset PAH has its own unique traits which differ from adult-onset PAH in several important aspects including sex bias, disease etiology, clinical presentation, response to therapy, and survival time, with the prevalence of 15–50 cases per million people for adult-onset PAH. In addition, the higher prevalence of PAH among women than men in adult-onset PAH, which is about three- to fourfold, is not observed in childhood-onset PAH, suggesting less dependence upon sex-specific interacting factors in childhood-onset PAH (Barst et al., 2012; Zhu et al., 2018a; Zhu et al., 2019). Childhood-onset PAH often shows slightly higher mean pulmonary arterial pressure, more decreased cardiac output, and increased pulmonary vascular resistance than adult-onset PAH at diagnosis (Zhu et al., 2018a). If left untreated, patients with PAH often undergo the rapid progression, with a median survival of 2.8 years in adults and 10 months in children (Barst et al., 1999; Runo and Loyd 2003).

Etiologically, according to the Pediatric Pulmonary Hypertension Network (PPHN), nationwide Netherlands PH Service, TOPP (Tracking Outcomes and Practice in Pediatric Pulmonary Hypertension) registry, and REHIPED (*REgistro depacientes con HIpertensión Pulmonar PEDiátrica*), childhood-onset PAH has a higher proportion of idiopathic PAH (IPAH) and PAH associated with congenital heart disease (PAH-CHD), with PAH-CHD constituting nearly 30.6%–60% of children with PAH, and IPAH and heritable PAH (HPAH) comprising about 23%–53.1% and 5%–18.4%, respectively (van Loon et al., 2011; Barst et al., 2012; Abman et al., 2022; Cruz-Utrilla et al., 2022). In addition, according to the PPHN, the majority of PAH in children were characterized by Eisenmenger syndrome in 10%; systemic-to-pulmonary shunts, excluding Eisenmenger syndrome, in 26%; and post-operative PAH in 25% (Abman et al., 2022).

IPAH is a sporadic form of PAH with unknown etiology, which is usually diagnosed in its later stages due to nonspecific symptoms, and the prevalence rate of IPAH in children is lower than that in adults (about 2.1–4.4 cases per million *vs*. 5.9–25 cases per million) (Moledina et al., 2010; van Loon et al., 2011; Ivy et al., 2013). In the histopathology, adults with IPAH often have severe intimal fibrosis, plexiform lesions, and irreversible pulmonary vascular changes, while children with IPAH have more pulmonary vascular medial hypertrophy, less intimal fibrosis, and fewer plexiform lesions (Barst et al., 2011). The definition of PAH-CHD in children is various heart defects with specific hemodynamic profiles associated with different temporal evolution patterns of pulmonary vascular disease or with congenital maldevelopment of the pulmonary vasculature (van Loon et al., 2011). In addition, the prevalence rate for pediatric PAH-CHD is higher than that reported in adults (about 15.6–21.9 cases per million *vs*. 1.7–12 per million) (Humbert et al., 2006; Peacock et al., 2007; van Loon et al., 2011; Li et al., 2017). The mortality rate in PAH-CHD was similar to IPAH and HPAH within 5 years, all of which were about 12% and worse than those of adults (van Loon et al., 2011; Ivy et al., 2013; Abman et al., 2022). It should be noted that the PAH genetics community has found HPAH to comprise familial PAH (FPAH, PAH that occurs in two or more family members) and PAH without a family history in a clear genetic diagnosis. Therefore, patients classified as IPAH at diagnosis should be reclassified as HPAH when a causal genetic variant appears (Welch et al., 2021).

Great genetic burden has been observed in childhood-onset PAH, with at least 35% of PAH in children being attributed to rare deleterious genetic factors (Welch and Chung, 2020). Genetic studies have indicated that mutations of some genes involved in the transforming growth factor-β (TGF-β) pathway, including bone morphogenetic protein receptor 2 (*BMPR2*), activin receptor-like 1 *(ACVRL1*), and endoglin (*ENG*), are associated with childhood-onset PAH (Barst et al., 2011; Rosenzweig et al., 2019). To date, there are at least nine genes associated with childhood-onset PAH. Abnormalities in *BMPR2* are the most commonly identified mutations in children with PAH, in approximately 50%–80% of FPAH and 10%–40% of IPAH, with a pattern of autosomal dominant inheritance (Zhu et al., 2018a; Rosenzweig et al., 2019; Zhu et al., 2019; Ivy and Frank, 2021). The innovation of gene detection technology has helped to identify novel genes associated with childhood-onset IPAH, including T-box 4 (*TBX4*), SRY-related HMG box transcription factor (*SOX17*), mothers against decapentaplegic 9 (*SMAD9*), potassium two-pore-domain channel subfamily K member 3 (*KCNK3*), caveolin-1 (*CAV1*), and growth differentiation factor 2 (*GDF2*) (Chida et al., 2012; Garcia-Rivas et al., 2017; Zhu et al., 2018a; Morrell et al., 2019; Rosenzweig et al., 2019; Montani et al., 2021). In addition, pediatric patients are more likely to be affected by IPAH and HPAH, suggesting genetic factors play a significant role in the pathogenesis of pediatric PAH (Berger et al., 2012; Hansmann and Hoeper, 2013). Identification of genetic subtypes of PAH along with known biological functions of the genes would provide important data on the natural progression of disease and is beneficial to help improve risk stratification, treatments, correlations of the phenotype and therapeutic responsiveness, and prognosis. Combined with previous studies and PPHN, we reviewed and summarized the identified risk genes contributing to childhood-onset PAH with large amounts of literature evidence to date: *BMPR2*, *TBX4*, *SOX17*, *SMAD9*, *KCNK3*, *CAV1*, *GDF2*, *ACVRL1*, and *ENG* (Table1)*.* In this review, we also discuss the most promising *BMPR2* gene therapy and gene delivery approaches for childhood-onset PAH in the future.

**TABLE 1:** Summary of genes of childhood-onset PAH.

Gene	Rate of mutation (%)	Mean age of onset (years)	Types of mutation			PAH type		Refs
			LGD (%)	Damaging missense variant (%)	Others(%)	IPAH (%)	HPAH (%)	
*BMPR2*	6.3–43	10.1–14	47.1–100	14.3–52.9	0–14.3	0–60	40–100	Kerstjens-Frederikse et al. (2013), Levy et al. (2016), Zhu et al. (2018a), Eyries et al. (2019), Zhang et al. (2019), Zhu et al. (2019), Haarman et al. (2020), Abman et al. (2022)
*TBX4*	2.1–25	4.4–8.6	52.6–100	0–25	0–31.6	58.3–100	0–33.3	Kerstjens-Frederikse et al. (2013), Levy et al. (2016), Zhu et al. (2018a), Eyries et al. (2019), Galambos et al. (2019), Zhu et al. (2019), Haarman et al. (2020), Abman et al. (2022)
*SOX17*	1.9–6.3	4.8–7.8	33.3–60	33.3–40	0–33.3	11.1–40	0–11.1	Levy et al. (2016), Zhu et al. (2018b), Zhu et al. (2019)
*SMAD9*	0.9–2.1	2.5–7.5	50–100	0–50	0	0–100	0–50	Levy et al. (2016), Zhu et al. (2018b), Zhu et al. (2018b), Graf et al. (2018), Zhu et al. (2019), Abman et al. (2022)
*KCNK3*	0.8–6.3	5.5–8	0–33.3	50–100	0–33.3	0–50	50–100	Levy et al. (2016), Zhu et al. (2018a), Zhang et al. (2019), Zhu et al. (2019), Haarman et al. (2020), Abman et al. (2022)
*CAV1*	0.4–3	5	100	0	0	0	100	Levy et al. (2016), Abman et al. (2022)
*GDF2*	0.9–6.8	9–17.7	0–27.3	68.2–100	0–4.5	100	0	Levy et al. (2016), Graf et al. (2018), Wang et al. (2019a), Zhu et al. (2019), Abman et al. (2022)
*ACVRL1*	0.9–12.9	9–10.7	0–50	50–100	0	0–75	0–100	Levy et al. (2016), Zhu et al. (2018a), Eyries et al. (2019), Zhang et al. (2019), Zhu et al. (2019), Haarman et al. (2020), Abman et al. (2022)
*ENG*	1.2–5	-	40–72.7	18.2–60	0–9.1	-	-	Zhu et al. (2018b), Graf et al. (2018), Zhang et al. (2019), Abman et al. (2022)

*ACVRL1*, activin receptor-like 1; *BMPR2*, bone morphogenetic protein receptor 2; *CAV1*, caveolin-1; *ENG*, endoglin; *GDF2*, growth differentiation factor 2; *KCNK3*, potassium two-pore-domain channel subfamily K member 3; LGD, likely gene-disrupting variants (including premature stop-gain, frameshift indels, canonical splicing variants, and deletion of exons); PAH, pulmonary arterial hypertension; *SMAD9*, mothers against decapentaplegic 9; *SOX17*, SRY-related HMG box transcription factor 17; *TBX4*, T-box 4.

### BMPR2

The detrimental variants in *BMPR2* account for nearly 50%–80% of FPAH and 10%–40% of IPAH cases in children (Kerstjens-Frederikse et al., 2013; Levy et al., 2016; Zhu et al., 2018a; Eyries et al., 2019; Zhang et al., 2019; Zhu et al., 2019; Haarman et al., 2020; Abman et al., 2022). The carriers with *BMPR2* detrimental variants are always younger, and they have worse hemodynamic characteristics at first consultation. In addition, children with PAH and *BMPR2* mutations are unlikely to respond to vasodilators, and it is impossible for them to gain benefit from treatment of calcium channel blockade (Elliott et al., 2006; Rosenzweig et al., 2008).

The mature BMPR2 polypeptide harbors 1,038 amino acids including an extracellular signal peptide, a ligand-binding domain, a single-pass transmembrane domain, an intracellular catalytic kinase domain, and an atypically long cytoplasmic tail, which is a member of the TGFβ superfamily of signaling molecules (Liu et al., 1995; Deng et al., 2000; International et al., 2000). The TGF-β family participates in the control of numerous cellular functions and homeostasis, including proliferation, differentiation, apoptosis, and endothelial–mesenchymal transition (Good et al., 2015). TGF-β is of great importance in the respiratory system, and TGF-β regulates the synthesis of platelet-derived growth factors in endothelial cells, which plays an important role in the process of the vascular smooth muscle cell growth (Roberts and Sporn, 1989; Gore et al., 2014).

The variants of *BMPR2* in childhood-onset PAH include missense mutations resulting in the substitutions of amino acids and nonsense mutations, the insertions or deletions of small nucleotides leading to frameshift mutations, gene rearrangements, and splice-site mutations leading to premature protein truncation (Machado et al., 2015; Southgate et al., 2020). It seems that nonsense or frameshift mutations truncate the proteins and result in nonsense-mediated decay, while missense mutations lead to reservation of the mutative proteins in the endoplasmic reticulum or plasma membrane (Frump et al., 2013). Given missense mutations distribute broadly, nearly throughout the *BMPR2* exons, and the majority of them are included in key functional domains, especially the ligand-binding domain encoded by exons 2–3 and the highly conservative catalyzed kinase region functionally deciphered by exons 6–9 and 11. In addition, various studies confirming genetic variations in *BMPR2* have been limited to the exonic space (Machado et al., 2015; Southgate et al., 2020). Furthermore, *BMPR2* haploinsufficiency has been identified as the primary molecular mechanism underlying hereditary PAH.

### TBX4

The detrimental variants in *TBX4* account for about 2.1%–25% of childhood-onset PAH, and predominantly occur in IPAH cases (Kerstjens-Frederikse et al., 2013; Levy et al., 2016; Zhu et al., 2018a; Eyries et al., 2019; Galambos et al., 2019; Zhu et al., 2019; Haarman et al., 2020; Abman et al., 2022). *TBX4* was first suspected as a candidate risk gene of PAH due to the location on chromosome 17q23.1 to 23.2, where microdeletions were associated with severe neurodevelopmental delays and pulmonary hypertension (Ballif et al., 2010; Nimmakayalu et al., 2011). The rare deleterious *TBX4* variants have later been reported to have an association with small patella syndrome and PAH (Bongers et al., 2004; Kerstjens-Frederikse et al., 2013). The mean age of onset for the carriers of *TBX4* mutations in children with PAH is about 4.4–8.6 years old, which is younger than carriers with *BMPR2* mutations (Galambos et al., 2019; Zhu et al., 2019; Southgate et al., 2020). Furthermore, children with PAH who carry *TBX4* variants often associate with serious lung, bone, and heart developmental defects (Galambos et al., 2019). However, many PAH cases associated with *TBX4* follow a more benign course of disease than *BMPR2* carriers (Navas et al., 2016).

T-box 4 (TBX4) is a transcription factor incorporated in the T-box gene family members. It expresses in the lung and tracheal stroma and in the heart atrium. It plays an important role in limb development, lung growth, and branching. According to the animal models’ studies of the respiratory system development, TBX4 has been proven to interact with fibroblast growth factor (FGF)10 during lung growth and branching (Chapman et al., 1996; Sekine et al., 1999; Sakiyama et al., 2003; Arora et al., 2012).

The variants of *TBX4* in pediatric PAH include likely gene-disrupting (LGD) variants (including premature stop-gain, frameshift indels, canonical splicing variants, and deletion of exons), damaging missense variants, heterozygous loss-of-function *TBX4* mutations and in-frame deletion, and LGD variants (52.6%–100%) and damaging missense variants (0%–25%) as predominant mutations(Kerstjens-Frederikse et al., 2013; Levy et al., 2016; Vanlerberghe et al., 2017; Zhu et al., 2018a). The mutations in *TBX4* related to PAH in children are mainly concentrated in key T-box domains, often leading to impaired amino acid displacements or premature truncation of mutational transcripts (Arora et al., 2012; Kerstjens-Frederikse et al., 2013; Levy et al., 2016). The decreased expression lesions of FGF10 are due to the decrease of TBX4 activity, which is the inducer of bone morphogenetic protein (BMP) 4. The expressions of FGF10 and BMP4 are of great significance to activate the BMP pathway in normal lung development, with reports that *TBX4*-mutant embryos show failure of endothelial cells to form vessels (Naiche and Papaioannou, 2003; Sountoulidis et al., 2012).

Except for the aforementioned evidence, some reports indicate that the BMP pathway promotes differentiation, while the TGF-β pathway pushes the systems to a higher proliferative state. Decreased activation of the BMP pathway might occur owing to the decreased expression of FGF10 and BMP4 when there are *TBX4* mutations which can lead to abnormal lung development and disordered BMP/TGF–β/SMAD signaling pathways. The imbalance between the BMP pathway and TGF-β pathway—toward a higher multiplicative status—perhaps ultimately lead to PAH (Archer et al., 2010).

### SOX17

Based on the current literature, rare harmful variants in *SOX17* account for about 1.9%–6.3% of children with PAH, in particular with PAH-CHD (Levy et al., 2016; Zhu et al., 2018b; Zhu et al., 2019). The mean age of onset for *SOX17* variant carriers is younger than that of *BMPR2* variant carriers. Furthermore, variants of *SOX17* will increase the risk of other vascular endothelium-related diseases.

SOX17 is a transcription factor that promotes angiogenesis and interacts with mature endothelial molecular mediators to reduce the expression of SOX17 in endothelial cells and to restrict angiogenesis through Notch activation (related to the BMPR2 signaling pathway) (Lee et al., 2014; Kim et al., 2016; Hurst et al., 2017). *SOX17* is a highly restricted gene encoding Wnt/β-catenin and Notch signaling transcription factors during development, which is also involved in the development of endoderm, vascular endothelium, hematopoietic cells, and cardiomyocytes (Hudson et al., 1997; Alexander and Stainier, 1999; Sekine et al., 1999; Kanai-Azuma et al., 2002; Zhang et al., 2005; Kim et al., 2007; Liu et al., 2007). The endothelial destiny of CD34 progenitor cells is also determined by *SOX17* (Francois et al., 2010; Zhang et al., 2017).

The variants of *SOX17* in pediatric PAH include LGD variants, damaging missense variants, and heterozygous *SOX17* mutation, and LGD variants (33.3%–60%) and damaging missense variants (33.3%–40%) as the predominant mutations (Zhu et al., 2018b; Zhu et al., 2019; Wang et al., 2021; Welch et al., 2021). Genetic studies in mice showed that *SOX17* plays a considerable part in the right development and performance of the pulmonary vascular tree. Inactivation of *SOX17* in some specific endothelial cells contributes to arterial damage and embryonic death (Corada et al., 2013). Deletion of *SOX17* in mice causes disorders of pulmonary vascular morphogenesis, poor distal lung perfusion, and biventricular hypertrophy which further result in postnatal cardiopulmonary insufficiency and infant mortality (Lange et al., 2014). In addition, a mice endothelial lineage tracing study reports that transcriptional activation of *SOX17* through hypoxia-induced factor 1α upregulates cyclin-E1 and contributes to endothelial regeneration after injury of the lungs (Liu et al., 2019). Therefore, there are a variety of mechanisms of *SOX17* deficiency or deletion which lead to heart and lung developmental defects or impaired response to hypoxia injury.

### SMAD9

Rare deleterious variants in *SMAD9* lead to approximately 0.9%–2.1% childhood-onset PAH and predominantly occur in IPAH cases (Levy et al., 2016; Zhu et al., 2018a; Zhu et al., 2018b; Graf et al., 2018; Zhu et al., 2019; Abman et al., 2022). The mean age of onset for *SMAD9* variant carriers is younger than that of *BMPR2* variant carriers and is about 2.5–7.5 years old (Levy et al., 2016; Zhu et al., 2018a; Zhu et al., 2018b; Graf et al., 2018; Zhu et al., 2019; Abman et al., 2022).

*SMAD9* encodes SMAD9, an intracellular signal transducer of the TGF-β pathway (van den Heuvel et al., 2020). As a member of the BMP signal cascade, SMAD9 is located in the downstream in the BMPR2 pathway. Furthermore, SMAD9 is related to the nitric oxide biosynthesis process, the synthesis of various oxidoreductases controlling oxidative stress and homeostasis in the cells (Garcia-Rivas et al., 2017). As functional analyses proved, *SMAD9* mutation significantly reduced SMAD transcription activity and heterozygous *SMAD9* mutations perturbed microRNA (miRNA) processing (Nasim et al., 2011; Machado et al., 2015). Malfunction of the SMAD signal can affect the proliferation of smooth muscle cells and malfunction of SMAD9 can lead to the high proliferation phenotype, weakened BMP signal, and SMAD-mediated microRNA processing disorder, which further contribute to PAH (Yang et al., 2005; Drake et al., 2011). Interestingly, overexpression of wild-type SMAD9 corrects these defects (Drake et al., 2011; Drake et al., 2015).

### KCNK3

Rare deleterious variants in *KCNK3*, coding for two-pore-domain potassium channels which are expressed in pulmonary arterial smooth muscle cells, contribute to approximately 0.8%–6.3% childhood-onset PAH, and predominantly appear in FPAH. The mean age of onset for *KCNK3* carriers is about 5.5–8 years old (Levy et al., 2016; Zhu et al., 2018a; Zhang et al., 2019; Zhu et al., 2019; Haarman et al., 2020; Abman et al., 2022).

It is membrane potential that plays a crucial part in pulmonary artery muscle cell (PASMC) contraction, and dysfunction of potassium channels has been implicated in the pathogenesis of PAH. *KCNK3* encodes an outwardly rectifying K^+^ channel that is sensitive to extracellular pH changes. The high expression of *KCNK3* is confirmed in PASMCs of rats, rabbits, and humans. The normal function of this channel is to induce the leakage K^+^ current, to keep the resting membrane potential, and to contribute to the regulation of the pulmonary vascular tone (Olschewski et al., 2017). The K^+^ channel is activated to induce K^+^ outflow, membrane hyperpolarization, and vasodilation (Olschewski et al., 2006; Olschewski, 2010). Genetic and electrophysiological studies suggest that *KCNK3* mutation, leading to downregulation of K^+^ channels, is a rare genetic cause of PAH (Ma et al., 2013). The decrease in activity in the PASMC K^+^ channel can contribute to cell multiplication, anti-apoptosis, and vasoconstriction, which ultimately leads to the remodeling of vessels (Le Ribeuz et al., 2020).

### CAV1

Rare deleterious variants in *CAV1*, encoding a specific protein which is essential for the integrity of the plasma membrane and the production of nitric oxide, are related to a small number of PAH cases, and contribute to approximately 0.4%–3% childhood-onset PAH. The mean age of onset for *CAV1* carriers is about 5 years old (Levy et al., 2016; Zhu et al., 2018a; Zhang et al., 2019; Zhu et al., 2019; Abman et al., 2022).

Caveolae, a specific protein in the membrane, encoded by *CAV1*, are enriched in mesenchymal cells and lung endothelial cells and are essential for mediating the cascade reaction of TGF-β, G protein, and nitric oxide signal in PAH (Maniatis et al., 2008). Abundant expression of *CAV1* is demonstrated in adipocytes, endothelial cells, and fibroblasts (Quest et al., 2004). Truncating mutation, *de novo* mutation, and frameshift mutation have been described in *CAV1*-associated PAH (Southgate et al., 2020). Frameshift mutations of *CAV1* in PAH cases, revealed by functional analysis, can cause the mutant protein to retain in the endoplasmic reticulum, which is accompanied by the retention of wild-type protein morphology, and furthermore results in significant damage to cell membrane caveolae assembly (Copeland et al., 2017). Furthermore, truncating mutation in *CAV1* causes the excessive phosphorylation of SMAD1, SMAD5, and SMAD9, which leads to the decrease in the antiproliferative function of caveolin-1, thus demonstrating that functional gain SMAD may be one of the potential molecular mechanisms of *CAV1*-associated PAH (Marsboom et al., 2017). In addition, BMPR2 is located in the cell membrane caveolae and interacts directly with the caveolin-1 in different cellular types, consisting of smooth muscle cells of vessels; therefore, the mutations in *CAV1* may also cause PAH by interfering with TGF-β signaling (Hartung et al., 2006; Wertz and Bauer, 2008).

### GDF2

Rare deleterious variants in *GDF2* contribute to approximately 0.9%–6.8% childhood-onset PAH (Levy et al., 2016; Graf et al., 2018; Wang et al., 2019a; Zhu et al., 2019; Abman et al., 2022). Nevertheless, progenitor-specific factors are likely to work in PAH, with *GDF2* being a dominant gene in Asian childhood-onset PAH patients compared to European patients (Wang et al., 2019a; Welch and Chung, 2020; Welch et al., 2021). Furthermore, *GDF2* has a strong association with IPAH cases and has been identified as one of the leading disease-associated genes in Asian childhood-onset PAH and is secondary to *BMPR2* (Wang et al., 2019a).

Expressed principally in the liver, GDF2 is a constituent secreted into circulation. GDF2 plays an important role in regulating vascular biology and angiogenesis by binding to ACVRL1 and BMPR2, and the optimal circulating GDF2 level is crucial for maintaining the resting state of pulmonary vessels. GDF2 is one of the members of the BMP subgroup of the TGF-β superfamily proteins, and its active form in circulation is essential for regulating vascular tension (Du et al., 2003; David et al., 2008). The PAH-specific mutations in *GDF2* include frameshift mutations, missense mutations, and nonsense mutations, and these mutations may inhibit biosynthesis and/or secretion of GDF2, leading to a decrease in GDF2 levels in the circulation in PAH patients which may be a cause of PAH (Wang et al., 2019a; Southgate et al., 2020). Furthermore, the results of cell experiments demonstrate pre-treatment of WT GDF2-conditioned medium protects PAECs from apoptosis induced by tumor necrosis factor-α, while these mutants are unable to prevent PAEC apoptosis (Wang et al., 2019a).

### ACVRL1

According to the previous studies, rare deleterious variants in *ACVRL1* are expressed in approximately 0.9%–12.9% childhood-onset PAH (Levy et al., 2016; Zhu et al., 2018a; Eyries et al., 2019; Zhang et al., 2019; Zhu et al., 2019; Haarman et al., 2020; Abman et al., 2022). The pediatric studies reveal a higher carrier frequency of *ACVRL1* variants in Asian childhood-onset PAH than that of predominantly Europeans (6.1%–12.9% *vs*. 0.9%–2.6%) (Chida et al., 2012; Zhu et al., 2018a; Zhang et al., 2019; Zhu et al., 2019; Haarman et al., 2020). Studies also demonstrate that *ACVRL1* has exome-wide extensive mutation enrichment in IPAH cases. Furthermore, *BMPR2*, *GDF2*, and *ACVRL1* have been identified as the top three disease-related genes in Asian childhood-onset PAH (Wang et al., 2019a). PAH patients harboring *ACVRL1* mutations have earlier onset, faster disease progression, and are younger at death than those with *BMPR2* mutations (Girerd et al., 2010).

Studies showed that most mutations in *ACVRL1* in PAH cases are missense mutations (Machado et al., 2009). Related literature data confirm that *ACVRL1* mutation carriers carrying PAH have dominant mutations in exon 10, especially in NANDOR boxes, which are essential for the regulation of TGF-β signaling (Harrison et al., 2005; Girerd et al., 2010). *ACVRL1* encodes the BMP receptor, ACVRL1, whose expression is mainly restricted to the vascular and lymphatic endothelia (Seki et al., 2003; Niessen et al., 2010). The disruption of the receptor complex transport was the main cause of the vessel phenotype of PAH deciphered by the functional studies using *ACVRL1* mutants (Harrison et al., 2003; Harrison et al., 2005). Further studies show *ACVRL1* mutants of PAH are related to the downregulation of SMAD1/5/9 signaling and confirm that the *ACVRL1* mutants have negative effects on SMAD1/5 function (Piao et al., 2016). Therefore, *ACVRL1* mutations may mainly impact the regulation of the TGF-β signalization pathway. TGF-β signaling pathway dysfunction could promote dysfunction and proliferation of endothelium and/or smooth muscle cells in pulmonary vessels (Trembath et al., 2001).

### ENG

Rare deleterious variants in *ENG* are seen more rarely and contribute to approximately 1.2%–5% childhood-onset PAH (Zhu et al., 2018b; Graf et al., 2018; Zhang et al., 2019; Abman et al., 2022). The mutations in *ACVRL1* or *ENG* genes primarily lead to PAH associated with hereditary hemorrhagic telangiectasia (HHT), which is a rare autosomal dominantly genetic disease. HHT is caused by telangiectasia enzymes and malformations of arteriovenous, including malformations of pulmonary arteriovenous. Due to different lesion sites, clinical manifestations of malformations of pulmonary arteriovenous are varied, which are primarily brought about by default in type I receptor ACVRL1 and default in type III accessory receptor ENG (Girerd et al., 2010; Ma and Chung, 2017; Southgate et al., 2020).

ENG is one of the members of the TGF-β signaling family and is expressed as the disulfide bond stable homodimer membrane-anchored proteoglycan, which is a marker of the proliferating endothelium. ENG is also an endothelial-specific receptor of BMPs, which lacks the intracellular kinase activity and acts as a co-receptor in complex with ACVRL1 (Saito et al., 2017). ENG shedding releases a soluble form of ENG through proteolytic enzyme cleavage of the extracellular domain of the receptor (Venkatesha et al., 2006; Bernabeu et al., 2009). ENG has been typified as a secondary receptor of the TGF-β–ACVRL1 signal in endothelial cells, which is crucial for regulating endothelial proliferation and migration (Jin et al., 2017; Sugden et al., 2017).

BMP9–BMP10 heterodimer is the high-affinity ligand of a receptor complex including ACVRL1, ENG, and BMPR2 (David et al., 2007; Scharpfenecker et al., 2007; Tillet et al., 2018). When BMP9/10 is activated, the ACVRL1–ENG–BMPR2 receptor complex phosphorylates SMAD1/5/9 and induces the expression of growth factors such as fibroblast growth factor 2 and platelet-derived growth factor B^39^. Dimers of phospho-SMAD1, phospho-SMAD5, or phospho-SMAD9 associate in a trimeric complex with SMAD4 and translocate into the nucleus of the endothelial cell where they bind to BMP-responsive elements on the promoters of target genes to either enhance or repress their expression (Garcia de Vinuesa et al., 2016; Goumans et al., 2018). The synthesis process of platelet-derived growth factors is regulated by TGF-β through the non-canonical pathway (C-terminal SRC kinase or mitogen-activated protein kinase) in endothelial cells, which can stimulate the growth of vascular smooth muscle cells (Roberts and Sporn, 1989). In addition, HHT is thus now considered by some researchers as a disease of the BMP9/10 pathway rather than a disease of the TGF-β pathway (Tillet and Bailly, 2014).

### *BMPR2* gene therapy and gene delivery systems

Although important advances in therapeutic management have shown that the quality of life and hemodynamic parameters of PAH have been improved, their benefits to survival and progression of PAH are very limited, as genes influencing PAH are unaffected. They change cell phenotypes and participate in the pathogenesis of PAH by improving the characteristics of hyperproliferation, promoting anti-apoptosis of pulmonary vascular cells, and regulating calcium equilibrium (Leopold and Maron, 2016; Rai et al., 2021). In the past decades, a series of new, mighty technologies enabled precise gene editing to accurately intervene in certain specific steps of the disease. These help to develop safe and effective PAH gene therapies to repair mutations and to recover or downregulate gene expression (Austin et al., 2017). These new regeneration methods have shown great potential in preclinical research studies, but large-scale, well-designed clinical trials are still necessitated for further evaluation of clinical efficacy and safety.

Although at least nine genes are associated with childhood-onset PAH nowadays, *BMPR2* has been identified by a body of evidence as the major risk PAH gene, and approximately 50%–80% of HPAH and 10%–40% of IPAH are thought to be driven by *BMPR2* mutations; thus, restoring BMPR2 expression to cure PAH seems to be the most reasonable and important approach of genetic therapy (Zhu et al., 2018a; Rosenzweig et al., 2019; Zhu et al., 2019; Ivy and Frank, 2021; Rai et al., 2021; Cruz-Utrilla et al., 2022). In addition, restoration of BMPR2 expression using gene therapy has shown promising results in animal models under research settings. Therefore, we summarized and discussed the advances of *BMPR2* gene therapy.

## Gene delivery systems

### Viral vectors

#### Retroviral and lentiviral vectors

The retroviruses and lentiviruses are single-stranded RNA viruses characterized by two long-terminal repeated sequences at either end of the genome and a packaging sequence, with the packaging capacity which can be up to 8 kb through a transgenic cassette inserted in a space generated by eliminating the natural viral components (*Gag*, *Pol*, and *Env*) (Szokol et al., 2004; Gandara et al., 2018). The viral RNA is converted into double-stranded DNA by a process of reverse transcription, which can be integrated into the chromosomal DNA of the host in a random way within the viral life cycle through the function of the viral integrase and can prolong and stabilize gene expression (Driskell and Engelhardt, 2003).

The first disadvantage of transfection for retroviral vectors in PAH is that retroviral vectors can only infect the target cells with the actively replicating property; however, the rate of cell proliferation in the lungs is low. In contrast, lentiviruses can transduce quiescent, post-mitotic cells with an efficient and economical transduction rate (Aneja et al., 2009; Lee et al., 2017). However, the requirement of the lentivirus-specific receptor, which is low and localized on the apical surface of the epithelial cells in the lungs, restricts the application of lentiviral vectors for the delivery of lung genes (Thomas et al., 2003; Waehler et al., 2007). A recent strategy to conquer this problem is to utilize pseudotyped lentiviral vectors derived from various origins, for example, Ebola, Sendai virus, influenza, and parainfluenza. Moreover, recent studies have found that G64-pseudotyped and Sendai virus-pseudotyped lentiviral vectors can transduce lung epithelial cells through the apical surface (Ferrari et al., 2007; Mitomo et al., 2010; Sinn et al., 2012). Several preclinical studies have shown that transduction with lentiviral vectors is safe; however, the random integration of viral DNA risks the insertional mutagenesis, the activation of cellular oncogenes, and the inactivation of tumor suppressor genes, which can further lead to tumor formation. Therefore, the long-term safety and efficacy of lentiviral vectors need further clinical studies (Anson, 2004; Mitomo et al., 2010; Alton et al., 2017).

#### Adenovirus vectors

The recombinant adenovirus (Ad) carries double-stranded DNA characterized by a natural tropism of the respiratory tract, an infection character for both quiescent and proliferating cells, relatively easy to produce and purify at a large scale, making them efficient vectors to transfer the lung gene. The double-stranded DNA of the adenovirus can escape from endolysosomal pathways and remains episomal; therefore, the risk of insertional mutagenesis in retroviral and lentiviral vectors can be avoided in Ad vectors, and the persistence of the therapeutic genes depends on the proliferation of the target cell. As a result, the expression of the transgene will be diluted if the cell proliferates with only one of the daughter cells containing the Ad genome (Driskell and Engelhardt, 2003; St George, 2003). The entry for Ad into the cells is through receptor-mediated endocytosis; however, the receptors are mainly located in the basolateral surface which limits the interactions between vectors and receptors due to the tight junctions of epithelial cells. Moreover, the highly immunogenic nature of Ad vectors can evoke the host immunity to Ad vectors, which limits the gene expression to 2–3 weeks and results in a low level for transfer of the therapeutic gene (Murata et al., 2005; Fausther-Bovendo and Kobinger, 2014). Therefore, the repeated administration on Ad vectors for effectively chronic expression of the therapeutic genes is unsuitable. Except for the aforementioned limitations, there is a body of evidence about the safety use of Ad vectors for lung gene therapy in preclinical rodent models (Chattergoon et al., 2005; Parker et al., 2013; Green et al., 2017).

In order to prolong the expression of transgenes and reduce the immune response of the host, second-generation Ad vectors have been developed by reducing the expression of viral antigens which is caused by deleting the E2 and E4 viral genes (Engelhardt et al., 1994; Chirmule et al., 1998; Cao et al., 2004). In experimental animals, these new vectors could minimize but not delete vector-induced inflammation response, which may be mediated by the antigenic capsid and the low-level expression of late viral genes (Chirmule et al., 1998). Currently, the “gutted/helper-dependent” adenovirus has been engineered in the attempt to further reduce Ad vector immunogenicity by eliminating all viral coding sequences. In experimental animal models, these new vectors showed longer transgene expression with a reduced immunity response of the hosts and the 36-kb packaging capacity (Jozkowicz and Dulak, 2005; Palmer and Ng, 2005; Brunetti-Pierri and Ng, 2008; Vetrini and Ng, 2010).

#### Adeno-associated virus vectors

Adeno-associated viruses (AAVs) are nonpathogenic, single-stranded DNA parvoviruses with self-replication defects, and AAV vectors are one of the most investigated gene transfer approaches. The recombinant AAV (rAAV) was developed by removing the *Rep* and *Cap* genes of the wild-type AAV and inserting the therapeutic transgene cassette, which is engineered as the gene therapy vector with the packaging capacity of up to 5 kb, a better transduction efficiency and long-term expression of the transgene in quiescent and proliferating cells and no risk of insertional mutagenesis because the rAAV persists in the nucleus as an episome (Kearns et al., 1996; Flotte, 2005; Li and Samulski, 2020). In addition, AAV vectors show low cytotoxicity and immunogenicity (Hernandez et al., 1999). Moreover, a body of evidence in experimental animal models has shown AAV can be used to transfer genes to the airway epithelium, alveolar epithelium, pulmonary vascular endothelium, and pleural mesothelioma (Han et al., 2008; Schultz and Chamberlain, 2008; Watanabe et al., 2010; Sondhi et al., 2017).

Nowadays, more than 100 AAV serotypes have been developed since the initially used two AAV serotypes, namely, 2 and 5, in order to improve the tropism of cells and tissues, magnify the therapeutic application fields, and reduce off-target effects (Lisowski et al., 2015). One of the disadvantages of this vector is that AAVs have little room to incorporate large genes because the genome is small. Recently, the strategy of using dual AAV vectors may have provided an approach to solving the limitation of packaging capacity (Halbert et al., 2002; Cooney et al., 2019). The AAV vector packaging capacity increases by developing a parvovirus chimera, with the rAAV genome packaged in the capsid of another parvovirus, such as human bocavirus (HBoV) or gorilla bocavirus (GBoV), and studies have shown the effective transduction efficacy of rAAV/HBoV and rAAV/GBoV in primary human airway epithelial cells, lung organoids, and ferret lungs (Yan et al., 2013; Yan et al., 2017; Fakhiri et al., 2019). Another drawback is that human beings can exhibit neutralizing antibodies to AAVs because they have already been exposed to various AAV serotypes, which further impairs the gene transfer (Schaffer and Maheshri, 2004). The solution strategy that has been put forward to solve this problem is the use of new site-mutated AAVs. Currently, studies have confirmed that these new site-mutated AAVs, such as site-directed mutagenesis (tyrosine to phenylalanine), could evade the immune response due to the pre-existing neutralizing antibodies and improve the AAV tropism and transduction efficiency as well (Wu et al., 2006; Zhong et al., 2008; Lompre et al., 2013).

#### Other viral vectors

A number of other viruses, such as polyomaviruses, vaccinia virus, baculovirus, and Sendai virus, have been used in lung gene therapy. Polyomaviruses, including simian virus 40 and John Cunningham virus, can deliver long-term expression of transgene in quiescent and proliferating cells by insertion into the host genome with relatively low immunogenicity. In addition, these vectors can be produced and purified in high titers (Vera and Fortes, 2004; Chang et al., 2011). Major concerns about these vectors are the limited packaging capacity of about approximately 2.5–5 kb and the risk of random integration (Grieger and Samulski, 2005).

Vaccinia virus vectors are another potential tool for lung gene transfer, whose packaging capacity can be up to 25 kb, with the characteristics of infection for most cells. Major concerns about vaccinia virus vectors are the induced cytopathic effects, immune responses, and the limited efficacy due to neutralizing antibodies to infections (Guo and Bartlett, 2004; Gregory et al., 2011).

Baculovirus expression vectors, which were initially used for recombinant protein expression, have the potential to serve as the vectors for gene therapy, as they can carry up to 38 kb DNA inserts and transfect various cell types without evoking immune responses in humans and without the risk of insertional mutagenesis. However, the major drawbacks of the vectors are the limited expression duration of the transgene, the requirement of entry through the basolateral membrane, and the rapid virus inactivation by serum complements, which should be further studied to optimize (Hu, 2006; Wang et al., 2020).

Vectors based on the Sendai virus have been developed for the transfer of lung gene with a natural tropism for respiratory epithelial cells and relatively high transduction efficiency (Inoue et al., 2004). However, the transgene expression is transient and the repeated administration can result in diminished gene expression. Currently, the strategy to solve these limitations is pseudotyping Sendai virus envelope proteins to lentiviruses for efficient transfection and sustained gene expression in lung cells (Griesenbach et al., 2012).

#### Non-viral vectors

Various non-viral vectors have been developed to deliver the therapeutic genetic material as naked DNA or RNA, including mRNA, siRNA, and microRNA, with the aim of enhancing the efficiency of entry into cells. Non-viral vectors can successfully overcome the constraints of the viral vectors, including the packaging capacity of the expression cassette, immunogenicity, biosafety risks, and the risk of insertional mutagenesis. RNA-based gene therapy does not require nuclear localization or transcription, whereas the DNA-based gene therapy requires translocation into the nucleus. An additional benefit is the negligible risk of genomic integration of the delivered sequence. The major limitations of non-viral vectors cannot be neglected, including the transient expression of therapeutic genetic material, the low tropism, and a low transfection efficiency *in vivo*, while they have significant advantages (Al-Dosari and Gao, 2009; Hill et al., 2016; Durymanov and Reineke, 2018).

Liposome reagents are a class of heterogeneous compounds prepared by combining phospholipids with fatty acid tails and hydrophilic head groups. Liposomes binding to plasmids have been widely used to enhance the internalization of the genetic material into the cytoplasm by endocytosis (Allen and Cullis, 2013; Rezaee et al., 2016). There are two extensively studied classes of liposomal vectors, anionic liposomes, and cationic liposomes. The anionic liposomes cannot directly connect to DNA; thus, DNA must be encapsulated inside the aqueous solution of the vesicle, which limits the packaged size of DNA and must require receptor-mediated endocytosis. The cationic liposomes with a positive charge binding to DNA by electrostatic interaction have larger packaging capacity than anionic liposomes. Currently, strategies to ameliorate liposomes are to permit targeting specific cell types because the liposomes adhere to cells non-specifically and further undergo endocytosis (Gallego et al., 2019).

Nanoparticles comprise the nucleic acid complexed with other materials, such as lipid or polymers including peptides or polysaccharides (Yin et al., 2014). Solid lipid nanoparticles maintain a solid state at physiological temperatures, can be prevented from nucleic acid degradation by the nucleases, and are often used to deliver siRNA (Sondhi et al., 2017). Polymer-based nanoparticles are more stable compared to lipids or liposomes, including natural or synthetic cationic polymers compounded with DNA. Due to higher efficiency of synthetic polymers, such as polyethyleneimine (PEI) or polyethylene glycol (PEG), they are often used to transfer genes despite the risk of cytotoxicity (Yin et al., 2014). Lipid nanoparticles (LNPs) are another fast and highly efficient method of transfecting cells by combining lipid-based components with siRNA or modRNA, and the combination of organic and aqueous components induces hydrophilic–hydrophobic interactions leading to nanoparticle formation (Reichmuth et al., 2016). In addition, the lipidoid components can be optimized by combinatorial chemistry to achieve higher stability of LNPs (Turnbull et al., 2017). However, the expression is transient, and the study showed that expression dropped to almost negligible levels in the second week after administration, which limits this method to the indications for feasible repeated administration or the preferred transient expression (Turnbull et al., 2016) (Table 2).

**TABLE 2:** Summary of vectors for gene transfer.

Vector	Transduction cell	Transduction efficiency	Expression	Packaging capacity	Genomic integration	Immunogenicity	Refs
Retrovirus vector	Proliferating cells	Low	Long-term expression	8 kb	Genomic integration	Moderate immunogenicity	Driskell and Engelhardt (2003), Thomas et al. (2003), Szokol et al. (2004), Waehler et al. (2007), Aneja et al. (2009), Lee et al. (2017), Gandara et al. (2018)
Lentivirus vector	Proliferating and quiescent cells	Low	Long-term expression	8 kb	Genomic integration	Moderate immunogenicity	Driskell and Engelhardt (2003), Thomas et al. (2003), Szokol et al. (2004), Waehler et al. (2007), Aneja et al. (2009), Lee et al. (2017), Gandara et al. (2018)
Ad vector	Proliferating and quiescent cells	High	Transient expression	4–5 kb	No genomic integration	High immunogenicity	Driskell and Engelhardt (2003), St George (2003), Chattergoon et al. (2005), Murata et al. (2005), Parker et al. (2013), Fausther-Bovendo and Kobinger (2014), Green et al. (2017)
AAV	Proliferating and quiescent cells	High	Long-term expression	5 kb	No genomic integration	Moderate immunogenicity	Kearns et al. (1996), Hernandez et al. (1999), Schaffer and Maheshri (2004), Flotte (2005), Han et al. (2008), Schultz and Chamberlain (2008), Watanabe et al. (2010), Sondhi et al. (2017), Li and Samulski (2020)
Nanoparticles	Proliferating and quiescent cells	High	Transient expression	>10 kb	No genomic integration	Low immunogenicity	Yin et al. (2014), Reichmuth et al. (2016), Turnbull et al. (2016), Sondhi et al. (2017), Turnbull et al. (2017)

AAV, adeno-associated virus; Ad, adenovirus.

## CRISPR/Cas9 as a gene-editing tool

The focus of gene therapy is to express exogenous DNA to cure diseases caused by gene mutations. CRISPR/Cas9 is a newly emerging, promising gene-editing technology which uses guide RNAs that have complementary sequences to the target site, which attempts to edit and repair mutated genes *in vivo*, insert new genes, or delete unwanted ones. Gene editing depends on homology-targeted repair or non-homologous DNA end joining, using the cellular mechanism of double-strand breaks in genomic DNA (Sondhi et al., 2017). The formation of the heterologous double-stranded complexes between guide RNA and DNA allows Cas9 nuclease to cleave, making it a simple and effective tool for gene editing (Alapati and Morrisey, 2017). The main drawback of this tool is the need for an adjacent protospacer motif and NGG sequence (Zhang et al., 2014). In addition, CRISPR/Cas9 can be used to regulate the reversible epigenetics of targeted genes. Enzyme-inactivated Cas9 can fuse with activator or repressor domains to activate or silence the target gene expression, respectively. Furthermore, CRISPR/Cas9 can be used for base editing. By fusing the inactivated Cas9 with other deaminases, a nitrogen-containing base can be replaced without producing a double-strand break (Maeder et al., 2013; Qi et al., 2013; Komor et al., 2016).

Preclinical studies have evaluated the therapeutic effects of CRISPR/Cas9 in a number of diseases, including congenital genetic lung diseases, infectious diseases, lung cancer, and immune diseases (Alapati et al., 2019; Ferdosi et al., 2019; Mohammadzadeh et al., 2020; Nair et al., 2020). Moreover, a recent study has reported that CRISPR/Cas9 can edit *Bmpr2* in rats (Kabwe et al., 2022). In addition, a clinical trial including 12 patients with metastatic non-small-cell lung cancer reported the clinical safety for the use of CRISPR/Cas9 gene-edited T cells targeting the PD-1 (Cyranoski, 2016). CRISPR/Cas9 holds tremendous potential, but further studies are needed to ensure the specificity of cleavage sites, avoid unexpected off-target effects, improve the efficiency of nuclease delivery, and guide RNAs to target cells.

### *BMPR2* gene therapy

In 2007, Reynolds and others demonstrated that in rodent experimental models, *Bmpr2*-containing Ad vector targeting pulmonary vascular endothelial cells could alleviate hypoxic pulmonary hypertension (Reynolds et al., 2007). The investigators surprisingly found that transfer of the *Bmpr2* gene can tremendously increase the BMPR2 expression, which increases Smad1/5/8 signaling and reduces the Smad2/3 signaling pathway, and significantly alleviate right ventricle hypertrophy and systolic blood pressure, mean pulmonary arterial pressure, and the hypoxia-induced vascularization (Reynolds et al., 2007). In 2016, other investigators used intravenous injection of Ad-mediated endothelial *Bmpr2* gene therapy to reverse PAH in mice carrying a *Bmpr2* mutation, as shown by reduced right ventricle systolic pressure and hypertrophy. In addition, they also found that the effects of BMPR2 regulation on non-SMAD signaling increased phosphoinositide-3 kinase, reduced phosphorylated-p38-mitogen-activated protein kinase, and upregulated nitric oxide production in the microvascular endothelial cells, which were related to relief of PAH, such as the decrease in right ventricular, mean pulmonary artery pressure and Fulton Index (Feng et al., 2016; Harper et al., 2016). Meanwhile, two other studies used the same monocrotaline-induced PAH model to explore the effects of *Bmpr2* gene therapy but found inconsistent results. One study found the intratracheal delivery of an Ad vector containing the *Bmpr2* gene did not cure the disease, despite the good distribution of the gene in the arteriolar network. Another study showed that intravenous injection of Ad-mediated endothelial *Bmpr2* gene therapy significantly alleviated right ventricular hypertrophy and pulmonary artery pressure (McMurtry et al., 2007; Harper et al., 2016). The different results may suggest the importance of targeting the endothelium for *BMPR2* gene therapy.

However, Ad vectors in these study limit their clinical use due to the risks of insertional mutagenesis reasons and the inflammatory response (Driskell and Engelhardt, 2003; Lee et al., 2017). Recent advances in the gene delivery technology develop new AAV vectors (Daya and Berns, 2008; Naso et al., 2017). At present, there are many AAV variants that show specific tropism characteristics, and moreover, the delivery of AAV-based gene therapy has high, long-term transduction and limited immunogenicity in experimental animals, showing a good application prospect (Mingozzi and High, 2013; Watanabe et al., 2018; Wang et al., 2019b; Bisserier et al., 2020). Currently, this strategy is investigated in preclinical studies of pulmonary hypertension.

## Challenges and future directions of pulmonary gene delivery in PAH

PAH is a fatal disease, and there is a growing interest in human gene therapy. The lung is an attractive tissue for gene therapy interventions because various gene delivery strategies, including intranasal, intravenous, intratracheal instillations, or aerosols, have been used for various lung diseases (Weiss, 2002). There is substantial evidence that preclinical PAH gene therapy successfully restores gene expression defects, corrects dysfunctional pathways, improves pulmonary vascular remodeling, inhibits disease progression, and reverses established diseases (Zhao et al., 2006; Reynolds, 2011; Katz et al., 2019). Intratracheal administration *via* aerosol delivery to the lungs, including intratracheal instillation or inhalation with or without bronchoscopy, showing low endonuclease activity, minimizing systemic side effects, and avoiding liver metabolism and liver absorption, serves as one of the favorable gene transfer pathways (Auricchio et al., 2002; Henning et al., 2010; Katz et al., 2019).

There are some advantages to intravenous administration of non-viral vectors entering the lung, including an almost unlimited packaging capacity, safe application, low immunogenicity, and the ability to simultaneously package both RNA species and DNA. However, various non-viral vectors are less efficient in gene transfer and provide only transient gene expression, and this obstacle can be overcome by repeated injections (Ramamoorth and Narvekar, 2015; van Haasteren et al., 2018). The insertion of tissue-specific DNA nuclear input signals found in some promoter sequences or the combination with the targeting peptide portion showed precise and enhanced cell targeting in the lung (Degiulio et al., 2010; Manunta et al., 2017). Driven by inflammatory response and hypoxia, PAH-lung vascular endothelial dysfunction and increased vascular permeability result in focal rupture of the endothelial basement membrane, and elevated vascular permeability contributes to enhanced nanoparticle accumulation in the diseased lung. Therefore, the nanoparticle-based gene therapy approach serves as a promising strategy for the treatment of PAH (Alton et al., 2015; Ramamoorth and Narvekar, 2015; Manunta et al., 2017).

Although virus vectors have good transduction efficiency, there are still some disadvantages to these vectors, such as the complex production methods, the immunogenicity and cytotoxicity, and the time-consuming and resource-consuming process that requires extensive experience to achieve consistent results for the concentration and titration of Ad, lentivirus, and AVV virus particles. Lentiviruses can randomly insert viral DNA into the host DNA, which leads to the risk of the insertional mutagenesis, the activation of cellular oncogenes, and the inactivation of tumor suppressor genes. Therefore, the extensive detection of these viruses in vector products and in patients is required by many regulatory agencies (Alton et al., 2017; Anson, 2004; Milone and O'Doherty, 2018). Ad vectors are highly effective in transduction of most cell types and tissues, but their capsids mediate potential inflammatory responses. Studies have shown that gene transfer with Ad vectors can lead to the induction of host immune response and apoptosis of vascular endothelial cells, indicating that pulmonary vascular endothelium exhibits specific antiviral immune activity that affects low gene transfer. In this respect, the production of helper-dependent Ad, characterized by more sustained gene expression, reduced immunogenicity, and eliminated capsid-mediated intense inflammatory responses is encouraging for effectively targeting airway basal cells *in vivo* (Murata et al., 2005; Brunetti-Pierri and Ng, 2006; Cao et al., 2018). AAVs have many benefits over the other vectors, including better transduction efficiency, long-term expression of the transgene, and relatively low immune responses and toxicities *in vivo*, and gene transfer with the AAV approach is currently among the most frequently used viral vectors systems. Drawbacks of AAV vectors include little room for AAVs to incorporate large genes, neutralizing antibodies to AAVs existing in human beings which further impair the gene transfer, and difficulties in manufacturing of pure AAVs (Schaffer and Maheshri, 2004; Moss et al., 2007; Daya and Berns, 2008; Katz et al., 2017). The discovery of new serotypes, an understanding of the tropism of each, and the subsequent generation of hybrid serotypes or capsid mutants can extensively improve the potential effectiveness of AAVs and will increase the effect of infecting only specific cell types in the lung. There have been many preclinical trials on successful delivery and transduction of AAVs for the safety and long-term efficacy of AAVs (Chamberlain et al., 2016; Verdera et al., 2020). Therefore, we believe that AAV-based therapy may be the most promising tool in the aforementioned approaches that can be safely applied to PAH studies.
